# The Difference in Zinc Concentrations Required for Induction among Metallothionein Isoforms Can Be Explained by the Different MTF1 Affinities to MREs in Its Promoter

**DOI:** 10.3390/ijms24010283

**Published:** 2022-12-23

**Authors:** Shoko Ogushi, Tomoki Kimura

**Affiliations:** Department of Life Science, Faculty of Science and Engineering, Setsunan University, 17-8 Ikedanaka-machi, Neyagawa 572-8508, Osaka, Japan

**Keywords:** metallothionein, zinc, transcription, MTF1

## Abstract

Metallothioneins (MTs) are cysteine-rich low-molecular-weight proteins that protect cells from heavy metal toxicity. MT1 and MT2 are considered ubiquitously expressed among the MT isoforms ranging from 1 to 4. These MT1 and MT2 transcriptions are regulated by metal regulatory transcription factor 1 (MTF1) binding to the metal response element (MRE) of the promoter, which is upregulated in response to zinc. The functional MT isoforms are MT1A, MT1B, MT1E, MT1F, MT1G, MT1H, MT1M, MT1X, and MT2A in humans, but these expressions were differently regulated. Here, MT1A was shown to be significantly less upregulated by zinc than MT1E, MT1G, MT1X, and MT2A. The poor responsiveness of the MT1A zinc was suggested to be due to the MRE sequence in the MT1A promoter region having a lower MTF1 binding affinity compared to the other isoforms. MT1A may be induced via pathways other than the MTF1–MRE binding pathway. These findings may help elucidate the differential regulation of MT isoform expression.

## 1. Introduction

For more than one and a half centuries, metallothionein (MT) has been a metal-binding protein with a molecular weight of approximately 6000–7000 found in the kidney cortex of horses [1]. It is found in many species and has high sequence and structural homology. Mammals have four isoforms (MT1–MT4). Of these, MT1 and MT2 are expressed in many tissues and play a central role in metal detoxification by trapping toxic metals via cysteine residues abundant in the molecules and inhibiting their binding to biocomponents [2]. MTs also play other roles in DNA damage, protection against oxidative stress, and carcinogenesis, as well as cancer therapy [3,4]. Some studies suggest that MTs act as storage sites for zinc ions under various conditions and play an important role in zinc transport [5]. Furthermore, humans have multiple MT isoforms, including MT1A, MT1B, MT1E, MT1F, MT1G, MT1H, MT1M, MT1X, MT2A, MT3, and MT4 [6]. It is reported that each isoform showed different expression patterns in tissues. They are believed to have resulted from gene duplication, and their amino acid sequences are very similar. In particular, the homology among MT1 isoforms and MT2A is >80%. While each isoform is speculated to play a different role, this is debated as the amino acid coding region sequence and the cysteine residue positions are highly preserved among the isoforms [7,8]. Metal responsiveness differs among isoforms, including the human cervical epithelioid carcinoma HeLa cells [9,10], the breast cancer cell line PMC42 cells [11], and the human urothelial cell line NHU cells [12]. Studies have been conducted to determine the relationship between MT expression patterns in human peripheral blood lymphocytes and metal exposure, and to explore the possibility as markers of metal exposure [13,14]. Emerging evidence shows that MT expression is not universal to all human tumors and may depend on tumor type, differentiation status, other environmental stimuli, and genetic mutations [4]. Reports that MT1A, MT1G, MT1X, and MT2A SNPs are involved in carcinogenesis, complications of atherosclerosis, and type 2 diabetes also support a shared role possibility for these isoforms [15,16]. Reports that certain MT isoforms interact with specific proteins also support the possibility of isoform role assignment [17,18,19]. Thus, although the functional differentiation of each MT isoform has been studied, including its relationship to pathogenesis, no conclusion has been reached that explains the need for isoform diversification.

**Figure 1:**
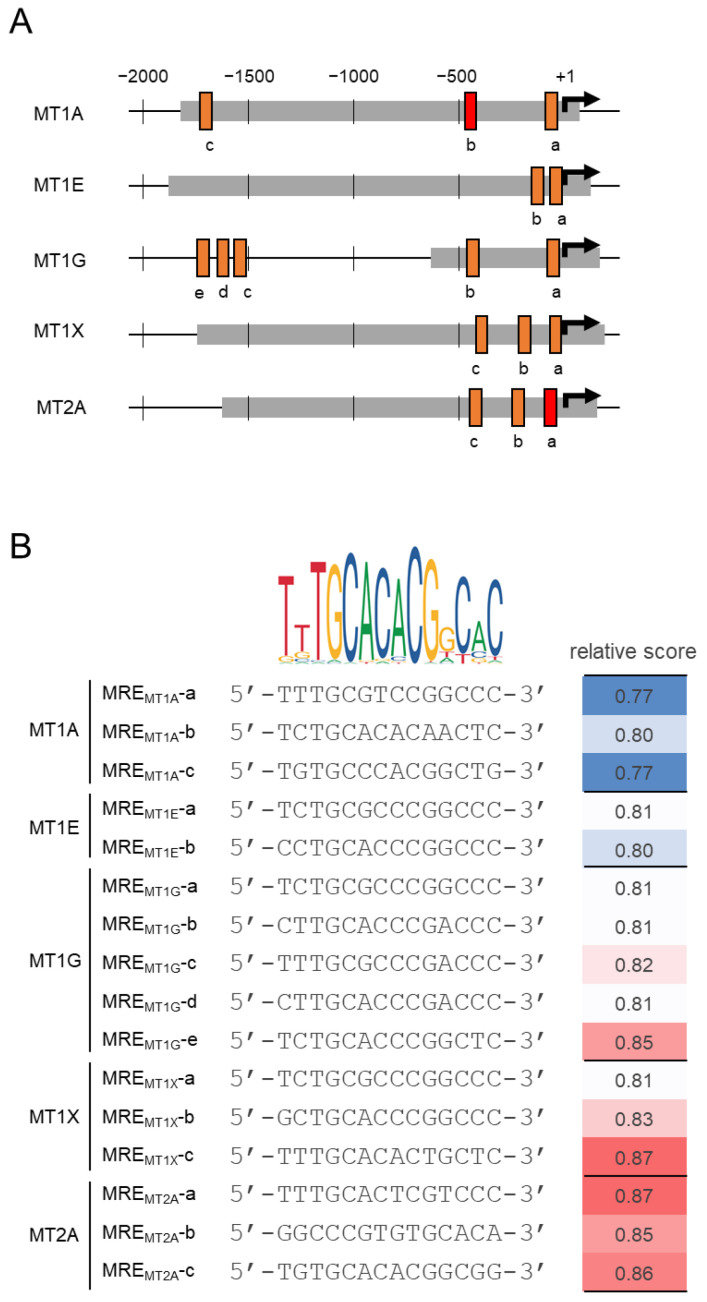
Schematic diagram of the MT promoter region. (**A**) The arrows indicate the transcription start sites. Orange and red squares indicate MREs (designated a–e, from the side closest to the transcription start site; the sequences in the red squares were used as competitor for EMSA assay) that is identified by the JASPAR search for MTF1 (Matrix ID: MA0863.1). The gray bold-lined area was cloned in the luciferase reporter vector. (**B**) Comparison between MRE consensus sequence and MRE sequences on each MT promoter. The relative scores obtained from the JASPAR search are shown in color scale on the right.

Regarding the MT induction mechanism, the metal regulatory transcription factor 1 (MTF1) is believed to play a central role in sensing heavy metal signals and transacting MT genes via the metal response element (MRE) [20]. Although the MT isoform coding regions are very homologous, the promoter region DNA sequences significantly differ from isoform to isoform, as do the MRE numbers and locations (Figure 1). Studies of differences in the transcriptional regulation between MT isoforms would help elucidate the role assignment, but little is known. In addition to MREs, the inactivation of CCAAT/enhancer-binding protein-α by the phosphatidylinositol 3-kinase signaling cascade suppresses MT expression in hepatocellular carcinoma [21]. Mouse MT1 promoters contain 12-*O* tetradecanoylphorbol-13-acetate responsive element, antioxidant responsive element, glucocorticoid responsive element, and IL-6 responsive element type II [22]. The myeloid master regulator transcription factor PU.1 represses MT1A and MT1G expression [23]. Epigenetic gene expression regulatory systems, such as CpG methylation and histone modifications, may be involved in MT gene expression. CpG site-specific regulation was previously reported to be present in mouse MT1 regulation [24]. It is possible that MT isoform-specific epigenetic gene expression control systems regulate these MT isoform expressions. For instance, some MT isoform expressions have been reported to be repressed during hematopoietic differentiation and that this repression is mediated by the epigenetic gene expression regulatory systems.

In this study, each MT isoform expression induction by zinc was examined, i.e., differential expression regulation by MTF1. Regulation by MREs was found to be nonuniform among the numerous MT isoforms and that MT1A is particularly poorly zinc-responsive. Electrophoresis mobility shift assay revealed that MRE sequences in the MT1A promoter region have a low binding affinity to MTF1.

## 2. Results and Discussion

### 2.1. MT Promoter Activation and Increase in MT Expression by Zinc Varies by Isoform

**Figure 2:**
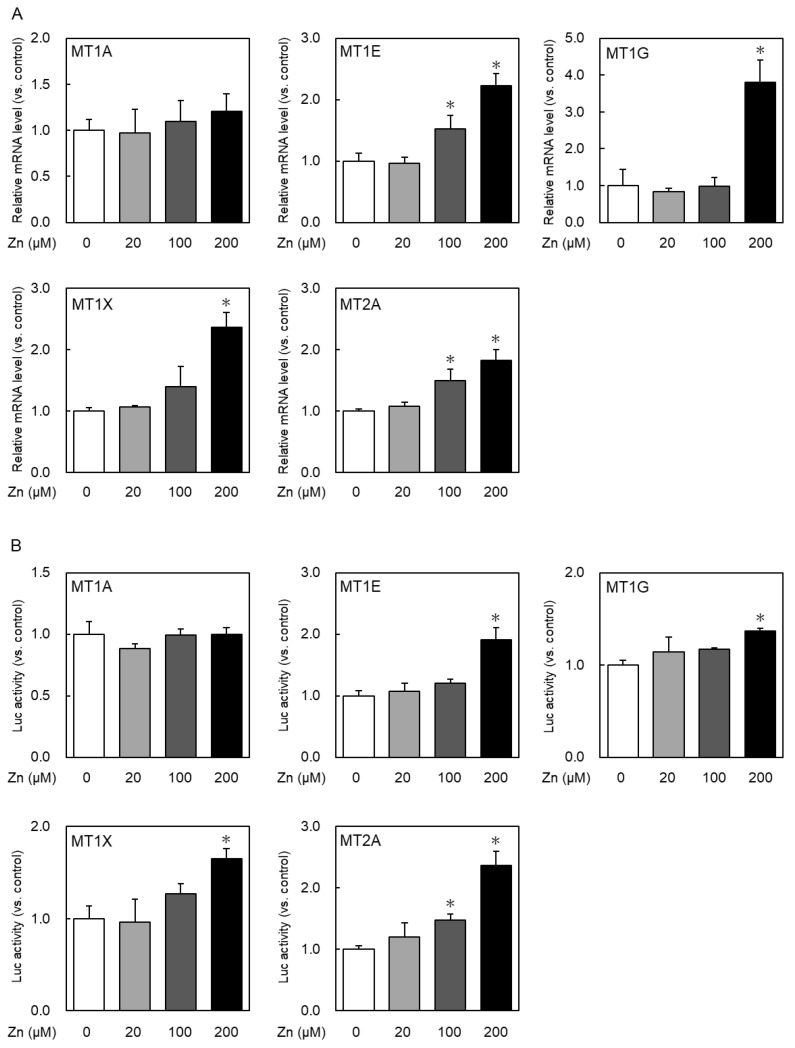
Each MT isoform expression after zinc treatment. (**A**) HeLa cells were treated with zinc at the indicated concentrations for 6 h, and the MTs mRNA expression was determined via real-time RT-PCR. (**B**) HeLa cells were co-transfected with luciferase reporter vector, pRL-SV40 vector for internal control, and pEGFP. At 24 h after transfection, the cells were treated with zinc at the indicated concentrations for 6 h, and luciferase activity was measured using the dual-luciferase assay system. pGL4.12-MT1A–1858/+23, pGL4.12-MT1E–1925/+43, pGL4.12-MT1G–636/+59, pGL4.12-MT1X–1879/+71, and pGL4.12-MT2A–1672/+55 were used as luciferase reporter vectors. * *p* < 0.05 indicates significant difference from the untreated group.

The MT expression level and MT promoter activity upon the addition of zinc were examined to elucidate the difference in induction by zinc for each isoform. HeLa cells were treated with 20–200-µM zinc for 6 h, and then the expression levels of the MT isoforms highly expressed in many tissues, MT1A, MT1E, MT1G, MT1X, and MT2A, were quantified (Figure 2A). Zinc increased the MT1E, MT1G, MT1X, and MT2A mRNA levels in a concentration-dependent manner. Contrarily, MT1A was not induced even when treated with 200 µM zinc. The low magnification of induction in MT1X and MT2A may be due to their high steady-state expression; MT1A, despite its low steady-state expression, showed no induction by zinc. In general, gene expression is regulated through several steps. The mRNA expression is mainly controlled by the transcription level, i.e., promoter activation and mRNA stability. Thus, luciferase reporter assays were performed to investigate whether the differences in MT1A mRNA levels were due to changes in promoter activity. Each MT isoform promoter-driven luciferase gene expression after 6 h of treatment with 20–200-µM zinc (Figure 2B) was compared. MT1E, MT1G, MT1X, and MT2A mRNA were increased in response to zinc treatment, but not MT1A mRNA. The promoter activity increased in a zinc concentration-dependent manner in MT1E, MT1G, MT1X, and MT2A, similar to the mRNA expression levels, whereas no increase was observed in only MT1A. Therefore, MT1A appears to be less easily induced by zinc than the other MT isoforms. Since the MT gene functions are performed by MT proteins, it is important to examine whether the amount of each MT isoform protein changes in the same manner as mRNA. However, at present, there is no antibody that can detect MT isoform proteins fractionally; therefore, only mRNA fractional quantifications were performed.

**Figure 3:**
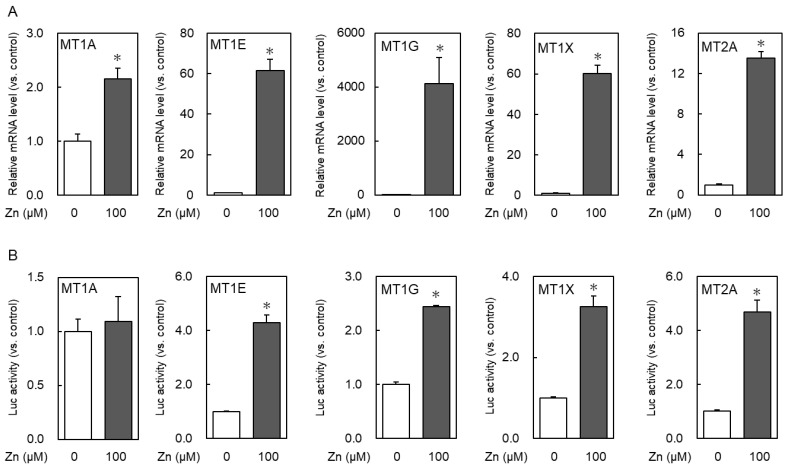
Each MT isoform expression after zinc treatment in 2-day zinc-deficient cultured cells. (**A**) HeLa cells, which were precultured for 2 days with zinc-deficient medium, were treated with zinc at the indicated concentrations for 6 h, and the MTs mRNA expression was determined via real-time RT-PCR. (**B**) The promoter activity after 6 h of zinc treatment was measured. The same vectors used in Figure 2 were used. * *p* < 0.05 indicates significant difference from the untreated group.

Next, whether the same difference in responsiveness was observed when zinc treatment was performed after 2 days of culture in a zinc-low medium was investigated, which was prepared using Chelex-treatment FBS. In MT1E, MT1G, MT1X, and MT2A, 100 μM zinc treatment significantly increased the mRNA levels and promoter activity compared with normal medium, whereas MT1A showed only a slight increase in mRNA expression levels and no change in promoter activity (Figure 3). The fold-induction of mRNA after zinc treatment differs greatly among isoforms. This may be due to the fact that the mRNA levels differ greatly among each MT isoform in the zinc-untreated group. These results indicate that MT1A, unlike other isoforms, is very poorly induced by zinc. In other words, its expression may not be MTF1-regulated.

### 2.2. MTF-1 Is Involved in the Regulation of MT1A Expression, as Are Other Isoforms

**Figure 4:**
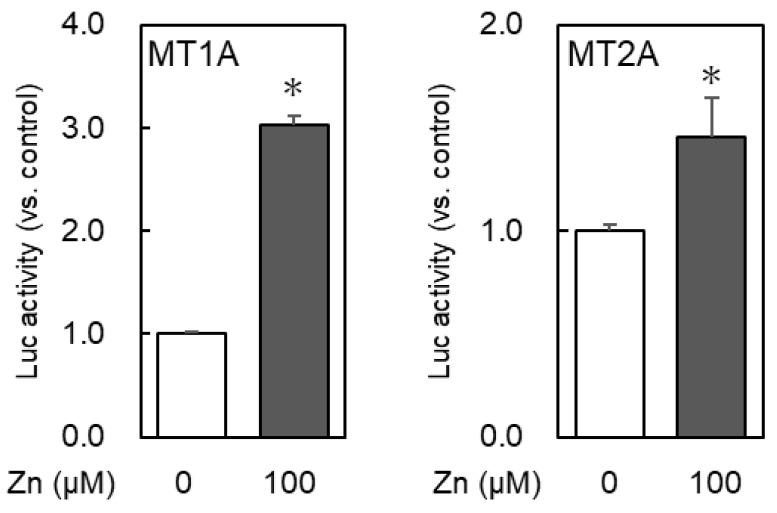
MTF1 co-expression effect on promoter MT1A and MT2A activity. HeLa cells were co-transfected with luciferase reporter vector, pRL-SV40 vector for internal control, and MTF1 expression vector. At 24 h after transfection, the cells were treated with zinc at the indicated concentrations for 6 h, and luciferase activity was measured using the dual-luciferase assay system. * *p* < 0.05 indicates significant difference from the untreated group.

Changes in the MT1A promoter activity after zinc treatment when the MTF1 expression vector was co-transfected and investigated to determine whether transcriptional MTF1 regulation also involves MT1A expression. MTF1 co-expression increased the MT1A promoter activity, which was further increased by zinc treatment. Furthermore, the MT1A promoter activity was increased in cells co-transfected with the MTF1 expression vector compared with non-transfected cells and was further increased by zinc (Figure 4). Thus, like other isoforms, MT1A was shown to be transcriptionally MTF1-regulated.

### 2.3. The MRE Binding Affinity in the MT1A Promoter to MTF1 Is Lower Than That of MRE in the MT2A Promoter

**Figure 5:**
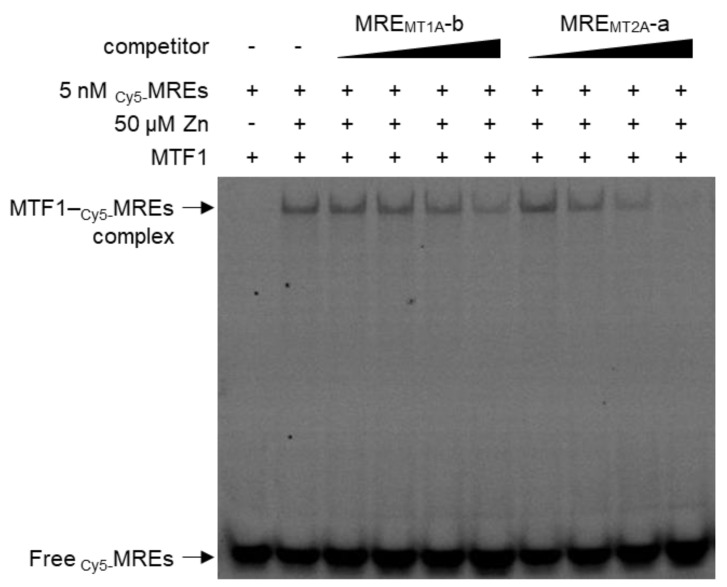
The MRE_MT1A_ and MRE_MT2A_ competitive effects on the binding reaction between MTF1 and MREs were examined via EMSA analysis. MTF1 protein was synthesized in a coupled transcription/translation reaction and incubated in a buffer containing 50 µM zinc. EMSA reactions were carried out containing competitive DNAs at 5, 100, 250, and 1000 nM. Binding of MTF1 to MREs was visualized by detecting Cy5-floorescence as low mobility MTF1–_Cy5_-MREs complex band.

MT1A, like other MT isoforms, is regulated by the transcription factor MTF1, but the extent of its induction widely varies. A possible reason for this is that MTF-1 is less likely to bind to the MRE sequence of the MT1A promoter. Competitive experiments were conducted by adding MRE sequences in the MRE_MT1A_ or MRE_MT2A_ to the binding reaction solution of _Cy5_-MREs and MTF1 to compare the MTF1 binding affinity to MREs in the MT1A and MT2A promoter regions (Figure 5). MREs is a sequence reported by Dr. Stuart in 1985 as an MRE sequence with high affinity to MTF1 [25]. Transcription factor Sp1 also often binds to MREs, but it is known that Sp1 does not bind to the MREs. Since MTF1 synthesized with TnT lysate, the effect of another transcription factors is expected to be minimal, but Cy5-labeled MREs were used to analyze the binding of MREs and MTF1. MRE_MT2A_ inhibited MTF1–_Cy5_-MREs complex formation at lower concentrations than MRE_MT1A_. Specifically, MRE_MT2A_ was more likely to bind to MTF1 than MRE_MT1A_. The MRE consensus sequence is shown in Figure 1B, which is illustrated in JASPAR and is 14 mer long; the MRE consensus sequence was previously shown as 5′-TGCRCNC-3′, which is 6-mer long, or 5′-NTGCRCNCgGCCc-3′ (small letters stand for poorly conserved bases), which is several bases further 3′ long [26]; MRE_MT1A_-b matches the 6-mer, but the gGCCc on the 3′ side does not match, which may explain the low affinity. On the other hand, it has been reported that MTF1 selects different MRE sequences at low and high zinc concentrations [27]. In the EMSA experiments, we added 50 µM zinc to MTF1, although it is possible that changing this concentration may change the selectivity. Such an investigation is warranted in the future. Another important experiment yet to be performed is to analyze which gene promoters MTF1 binds to by performing ChIP-seq with MTF1 antibody in cells exposed to various concentrations of zinc. In any case, our results indicate that MT1A is less likely to bind to MTF1 than other MT isoforms, such as MT2A, and is therefore less likely to be induced by zinc.

## 3. Materials and Methods

### 3.1. Cell Cultures and Cd Treatment

HeLa cells were sourced from RIKEN BioResource Center Cell Bank (Tsukuba, Japan), and Dulbecco’s Modified Eagle’s Medium (DMEM) (Nacalai Tesque, Inc., Kyoto, Japan) was used for culture supplemented with 10% heat-inactivated fetal bovine serum (Biowest, Nuaillé, France) and a mixture of antibiotic–antimycotic solution (Nacalai Tesque, Inc., Kyoto, Japan) at 37 °C under a 5% CO_2_ atmosphere. The cells were passaged every 2 or 3 days before cell seeding. Trypan Blue exclusion assays were used to measure cell viability and density [28]. The cells were seeded in a 12-well plate at a density of 6.0 × 10^4^ cells/well for the quantitative reverse transcription PCR and in a 24-well plate at a density of 3.3 × 10^4^ cells/well for the luciferase reporter assay. About 48 h after seeding, the cells were treated with zinc for 6 h. ZnSO_4_ was used for zinc treatment. Chelex^®^-100 chelating resin was purchased from Bio-Rad (Hercules, CA, USA). The cells were seeded and cultured in DMEM for the zinc-deficient culture and supplemented with 10% Chelex^®^-100-treated FBS (zinc-deficient medium) prepared according to the manufacturer’s protocols.

### 3.2. Quantitative Reverse Transcription PCR

RNA extraction reagent Isogen (Nippon Gene Co., Ltd., Tokyo, Japan) was used to isolate the total RNA from cells according to the manufacturer’s protocol. The High-Capacity cDNA Reverse Transcription Kit (Thermo Fisher Scientific, Inc., Waltham, MA, USA) with a random primer was used to convert complementary DNA from the extracted total RNA. The Eco Real-Time PCR System (Illumina, Inc., San Diego, CA, USA) with PCR reagents TB Green Premix Ex Taq (Tli RNase H Plus) (Takara Bio Inc., Kusatsu, Japan) or Premix Ex Taq (Probe qPCR) (Takara Bio Inc., Kusatsu, Japan) was used to perform qPCR. The primer and probe sequences were as previously reported [29]. All primer sets were purchased from Integrated DNA Technologies, Inc. (Coralville, IA, USA).

### 3.3. Plasmid Constructions

Flag-tagged human MTF1 cDNA was generated, and the expression vector pcDNA3.1-hMTF1_flag_ was constructed in the same manner as previously described [30]. The firefly luciferase reporter vector pGL4.12[luc2CP] was purchased from Promega (Madison, WI, USA). JASPAR (https://jaspar.genereg.net/, Matrix ID: MA0863.1 (accessed on 30 November 2022)), an open-access database storing manually curated transcription factor-binding profiles, was used to find putative MTF1 binding sites. The reporter vectors that contain each MT isoform’s promoter regions were constructed using primers as presented in Table 1 and with In-Fusion HD^®^ Cloning Kit (Takara Bio Inc.).

### 3.4. Luciferase Reporter Assay

After 24 h of cell seeding, various MT promoter reporter vectors were transfected. Transfection was performed according to the manufacturer’s protocol using transfection reagent FuGENE HD (Promega). In brief, the reporter vector, pRL-SV40 (Promega, Madison, WI, USA) and pEGFP or pcDNA3.1-hMTF1_flag_, was mixed at a 1:0.04:7.5 ratio in Opti-MEM medium (Invitrogen, Carlsbad, CA, USA). The plasmid mixture (0.5 µg per well) was mixed with FuGENE HD (1.5 µL per well) incubated for 15 min at room temperature, added to the cells cultured in a 24-well plate, and cultured for 24 h. The cells were then treated with zinc, incubated for 6 h, and then collected, and luciferase activity was measured. The cells were lysed in 1 × passive lysis buffer (Promega, Madison, WI, USA), whereas luciferase activity was measured using the Dual-Luciferase Reporter Assay System (Promega) and GloMax 20/20n luminometer (Promega).

### 3.5. Electrophoresis Mobility Shift Assay (EMSA)

The pcDNA3.1–MTF1flag and the TnT^®^ T7 Quick Coupled Transcription/Translation System were used to synthesize human MTF1 according to the manufacturer’s protocols (Promega). Cy5-labeled MREs (_Cy5_-MREs) were purchased from Integrated DNA Technologies, Inc., and a putative MRE within the MT1A promoter region (MRE_MT1A_-c) and MT2A promoter region (MRE_MT2A_-a), which has the highest relative score for MTF1 in each promoter, were purchased from Fasmac Co., Ltd. (Asugi, Japan). These sequences are presented in Table 1. MTF-1 (1 µL) was incubated at 37 °C for 15 min with 50 µM zinc in 10 μL 2 × buffer (24-mM HEPES, 120-mM KCl, 10-mM MgCl_2_, and 1-mM DTT) and 1 µL 1 mg/mL poly (dI-dC). Then, 1.0 µL of 0.1-µM _Cy5_-MREs and the indicated MRE_MT1A_ or MRE_MT2A_ amounts were added and incubated at 4 °C for 15 min. The mixture was separated from free _Cy5_-MREs and the MTF1–_Cy5_-MREs complex via electrophoresis on a 6% acrylamide gel. These _Cy5_-MREs were then visualized using the PharosFX System (Bio-Rad, Hercules, CA, USA).

### 3.6. Statistical Analysis

Data were analyzed for differences between multiple groups using Tukey’s test (with *p* < 0.05 considered significant) implemented in the PASW Statistics 18 package of SPSS (IBM, Armonk, NY, USA).

## 4. Conclusions

In the present study, the zinc responsiveness of MT1A was found to be significantly lower than that of other isoforms, and the MRE sequence in the MT1A promoter has a lower MTF1 affinity. The findings in this study suggest that the regulatory MT1A expression mechanism is poorly regulated by MTF1, unlike other isoforms. Further studies are warranted to determine the main pathway that induces MT1A expression, though it is possible that MT1A is mainly induced through pathways other than the MTF1–MRE binding pathway. Little is known about the differences in the roles between isoforms despite the fact that MTs are important proteins with many roles in biological defense. It is important to elucidate what environmental factors induce MT expression and through which pathways and whether the responsiveness to each pathway differs among isoforms. Further investigation is needed to explore the differences in expression regulation by isoforms and to understand the roles that they play.

## Figures and Tables

**Table 1:** Oligo DNA sequence used for EMSA and cloning.

		Sequence
Probe for EMSA	_Cy5_-MREs	5′-GATCCAGGGAGCTCTGCACACGGCCCGAAAAGTA-3′
	MRE_MT1A_-b	5′-CGGTGGCGGTTGCTCTGCACACAACTCGCTCGCTACCGCA-3′
	MRE_MT2A_-a	5′-CCGGGGCGGGGCTTTTGCACTCGTCCCGGCTCTTTCTAGC-3′
In-fusion cloning	MT1A-F	5′-GCTCGCTAGCCTCGAGTATTAGGGCAGGGAGGTG-3′
	MT1A-R	5′-TCTTGATATCCTCGAGAAGGCGCACGTGGAAGGC-3′
	MT1E-F	5′-CTCGGCGGCCAAGCTCTGCTCCCACAAAATCATC-3′
	MT1E-R	5′-CCGGATTGCCAAGCTACCAGTGAGACGAACAAGC-3′
	MT1G-F	5′-GCTCGCTAGCCTCGAGGAGGACCTGGACAAATGGG-3′
	MT1G-R	5′-TCTTGATATCCTCGAGGAGACTAGAGTTCCCAAGC-3′
	MT1X-F	5′-GCTCGCTAGCCTCGACACATGGCAAATGATCTATAA-3′
	MT1X-R	5′-TCTTGATATCCTCGATTCGAGGCAAGGAGAAGC-3′
	MT2A-F	5′-GCTCGCTAGCCTCGAGCAGGGTGGTCAAGAGGTG-3′
	MT2A-R	5′-TCTTGATATCCTCGAGGCTAGAGTCGGGACAGGT-3′

Underlines indicate the MRE consensus sequence. Double lines indicate the sequences homologous to the vector.
